# Bisphenol A and S in the Urine of Newborns: Plastic for Non-Food Use Still without Rules

**DOI:** 10.3390/biology10030188

**Published:** 2021-03-03

**Authors:** Valeria Bellisario, Enrico Cocchi, Roberta Tassinari, Giulia Squillacioti, Tiziana Musso, Stefano Sottemano, Michael Zorzi, Paola Dalmasso, Alessandra Coscia, Claudio Medana, Roberto Bono

**Affiliations:** 1 Department of Public Health and Pediatrics, University of Turin, 10126 Turin, Italy; enrico.cocchi@unito.it (E.C.); roberta.tassinari@unito.it (R.T.); giulia.squillacioti@unito.it (G.S.); tiziana.musso@unito.it (T.M.); stefano.sottemano@unito.it (S.S.); paola.dalmasso@unito.it (P.D.); alessandra.coscia@unito.it (A.C.); roberto.bono@unito.it (R.B.); 2 Health Statistics and Biometrics Residency School, University of Turin, 10126 Turin, Italy; 3 Center for Precision Medicine and Genomics, Department of Medicine, Division of Nephrology, Columbia University, New York, NY 10032, USA; 4 Unit of Mass Spectrometry, Department of Molecular Biotechnology and Health Sciences, University of Turin, 10126 Turin, Italy; michael.zorzi@unito.it (M.Z.); claudio.medana@unito.it (C.M.)

**Keywords:** BP neonatal exposure, BP non-alimentary contamination, human and childhood health, BP regulation

## Abstract

**Simple Summary:**

The aim of our study was to assess the effects of Bisphenols exposure on pregnancy and neonatal life. In this optic, we have: (a) determined Bisphenols concentration levels (Bisphenol A and Bisphenol S) in a group of newborns and their mothers, (b) identified factors, habits and devices possibly responsible for Bisphenols uptake, and (c) determined some possible health effect of Bipshenols exposure. The statistical analyses showed no significant correlations between maternal and neonatal Bisphenols concentration levels. In newborns, on the contrary, a positive correlation between pacifier use and Bisphenol S total and free concentration was detected. Beside, a significant correlation was also found between oral glucose administration and concentration levels of free Bisphenols A. Our study points to a central role of lifestyle, hospital procedures and neonatal devices in inducing Bisphenols exposure during perinatal period. This is the first report of Bisphenols contamination in newborns due to widely non-alimentary products destined for newborn care (glucose solution containers for Bisphenol A and the pacifiers for the Bisphenol S). Further studies are advocated to clarify both the impact of such other Bisphenols forms on human health and the potential Bisphenol A exposure sources during neonatal and childhood life.

**Abstract:**

The aim of the present study was to assess the effects of bisphenol (BP) exposure on pregnancy and neonatal life. We have (a) determined BP (BPA and BPS) concentration levels in a group of newborns and their mothers; (b) identified factors, habits, and devices possibly responsible for BP uptake; and (c) determined the effect of BP exposure. No significant correlations were detected between maternal and neonatal BP concentration levels. In newborns, positive correlations between pacifier use and BPS total (*p* = 0.04) and free BPS (*p* = 0.03) concentrations were detected. A significant correlation was also found between oral glucose administration and concentration levels of free BPA (*p* < 0.05). Our study points to a central role of lifestyle, hospital procedures, and neonatal devices in inducing BP exposure, especially during the perinatal period. This is the first report of BP contamination in newborns due to widely non-alimentary products designed for newborn care, such as glucose-solution containers for BPA and pacifiers for BPS. Further studies are advocated in order to clarify both the impact of other BP forms on human health and development, as well as potential BPA exposure sources during neonatal and childhood life.

## 1. Introduction

Bisphenols (BPs) have long been used for the production of polycarbonate plastics and epoxy resins [1]. BPs are well-known as harmful substances for human health, and they are usually ingested mainly through the diet, but BP intake can also occur by inhalation or dermal contamination.

Once they have entered the body, BPs exert estrogenic and/or oxidant activity [2,3], which is particularly harmful during pre- and neonatal life [4,5]. BPs are detoxified to inactive forms in the liver, primarily through conjugation and glucuronidation, then excreted in the urine within 2 to 6 h. Thus, the detection of conjugated BP forms is critical for determining BP risk exposure. In the glucuronidated form, BPs—particularly Bisphenol A (BPA) (their main representative member)—are inactive, whereas their free (unconjugated) forms, albeit basically unstable, promote biotoxicity through mild estrogenic activity [6]. BPs are prevalently found in urine (75–90%), but they are also traceable, albeit at lower concentrations, in other body fluids such as blood, breast milk, semen, cord blood, fetal serum, and placental tissue [7,8,9,10]. 

In this scenario, gestation appears to be a critical window of fetal exposure to BPs, as they trigger cellular responses even at very low doses [11], influencing sex- and gender-differentiation morphology or leading to immune hyper-responsiveness [12]. Infants appear to be particularly susceptible to the harmful effects of BPs [13], and prior studies have demonstrated that exposure to these chemicals can occur through both breastfeeding and skin contact with plastic devices (e.g., polycarbonate feeding bottles and pacifiers) routinely employed in neonatal intensive care units [11,12,14]. This harmful exposure is further exacerbated by the inefficient UDP-glucuronosyltransferase system in newborns, which is required for BP detoxification and not completely developed until ~2–3 months of age [14,15,16]. 

In 2015, following a public consultation on the harmful effects of BPA on human health [17], the European Food Safety Authority (EFSA) reduced the temporary tolerable daily intake (t-TDI) threshold of BPA from 50 to 4 µg/kg bw/day. Since then, plastics manufacturers have been striving to replace BPA with alternative compounds, including bisphenol S (BPS), its closest chemical relative. However, BPS has also recently been shown to exert genotoxic and biological activity similar to that of BPA [18,19]. These lines of evidence indicate that these BPA alternative compounds may also become a serious public health concern in the near future due to lack of regulatory limits [20,21].

Given these premises, the aim of the present study was to assess the modulation and possible health effects of BP exposure on perinatal and neonatal life. The final aim of this study was to investigate direct and indirect pathways and effects of BP exposure during pregnancy and the first days of life. Toward this end, we determined both BPA and BPS concentration levels in a group of newborn babies and their mothers. We particularly intended to focus our study on the identification of factors, devices, and behaviors responsible for high BP uptake in this sensitive population in the very first days of life. Finally, we evaluated if higher BP concentrations are correlated with specific hospital procedures or babies’ health status immediately after birth.

## 2. Material and Methods

### 2.1. Epidemiological Sample

The epidemiological sample was recruited from July 2016 to October 2017 by consulting the register of births of the Sant’Anna Gynecological Hospital (Turin, Piedmont Region), following these selection criteria: (1) full-term pregnancy (>37 GA-W-); (2) physiological pregnancy conditions; (3) no drugs or pharmacological treatment during pregnancy; (4) single babies (no twins) with Apgar scores > 5; and (5) healthy babies at birth (not admitted to the neonatal intensive care unit or in life-threatening conditions). Each adult subject was informed about the aim of this study and signed their written informed consent. Newborns were enrolled in the study upon written authorization from both parents. Sensitive data were replaced by anonymous identification codes to ensure full privacy of data. The local Ethics Committee of “A.O.U. Città della Salute e della Scienza” of Turin (22 October 2015, file No. CS/709) approved the study protocol.

### 2.2. Questionnaire

A standardized questionnaire (PRAMS questionnaire) [22] was administered to the mothers during hospitalization. Questions regarded individual, socio-demographic, and clinical characteristics such as smoking, diet, the mother’s working and lifestyle habits, especially in the last month of pregnancy, and education. Infant data, filled in by neonatologists, included gestational age and weight, sex, head circumference, length, the baby’s health status at the time of birth, and any medical check-ups. 

### 2.3. Biological Analysis

#### 2.3.1. Urine Sample Collection

A pool of fresh urine was collected from each subject, both for babies and their mothers. Infants’ urine samples were collected by means of a specific BP-free polypropylene bag (Urinocol Pediatric, B BRAUN, Milan, Italy) placed inside each newborn’s diaper from their birth to their third day of life, during hospitalization. Mothers’ urine samples were collected in glass tubes (Pyrex, CORNING, Corning, NY, USA) during hospitalization. All urine samples (infants and mothers) were then stored in glass mini-jars, pre-treated with methanol to reduce the risk of environmental contamination, and stored at −80 °C until analysis.

#### 2.3.2. Free BPA and BPS 

Two milliliters of urine was transferred in a tube pre-treated with methanol, vortexed, and acidified with HCl at pH 1. Subsequently, NaCl was added (salting-out process) together with 30 μL of a standard solution containing BPS-d8 and BPA-d16 with a final concentration of 0.10 and 0.05 mg/L, respectively. Next, 750 μL of chloroform and 500 μL of acetone were added for liquid–liquid extraction (LLE). Each sample was vortexed and then sonicated for 1 min. Finally, the sample was centrifuged at 1250× *g* for 20 min at room temperature, and the resulting supernatant was collected and transferred to a new pre-treated vial. LLE was repeated by adding 800 μL of chloroform. All of the supernatant was then brought to dryness by means of a nitrogen stream at room temperature and then resuspended in 100 μL of a solution composed of 5 mM ammonium acetate in ultrapure water (70%) and 5 mM ammonium acetate in acetonitrile (30%). 

#### 2.3.3. Total (Free + Conjugated) BPA and BPS

Four milliliters of fresh urine was thawed, vortexed, and aliquoted in two vials: 2 mL was used for the determination of free BP, while the other 2 mL was used for the determination of bisphenol-glucuronides after 12 h of incubation with 20 units of β-glucuronidase/arylsulfatase. 

#### 2.3.4. Conjugated BPA and BPS 

After 12 h of incubation with the enzyme β-glucuronidase/arylsulfatase, the same extraction procedure was performed as described above.

#### 2.3.5. Analysis

The instrumental analysis for both the free and conjugated procedures was carried out with a UPLC Shimadzu Nexera X2 System interfaced through an ESI source (Turbo Ion Spray™) to a Sciex 5500 QTrap mass spectrometer. The analytes were detected in negative ion mode. Concerning the LC set-up, the chromatographic column consisted of a Phenomenex (Bologna, Italy) Luna Omega C18 (1.6 μm, 100 mm × 2.1 mm), and the mobile-phase solvents for reverse-phase analysis were 5 mM ammonium acetate in water and 5 mM ammonium acetate in acetonitrile. The final pH value of both solvents was corrected to 7.5–8.0 by adding a few drops of 33% ammonia solution to ensure a more significant presence of BPA and BPS in deprotonated form. Concerning chromatographic gradient, the flow rate was set at 0.35 mL min^−1^. Mobile phases, initially consisting of 30% of 5 mM ammonium acetate in acetonitrile and 70% of 5 mM ammonium acetate in water, were held for 2 min then increased linearly to 100% organic solvent over 12 min, held there for 3 min, and finally brought back to the initial condition in 0.1 min. Moreover, 7 min of re-equilibration was necessary between samples. 

Moving to MS parameters, the drying gas (nitrogen) was set at 325 °C, 20.0 psi, and 10 L min^−1^; capillary voltage was set at 2000 V. Data acquisition was made in multiple reaction monitoring (MRM) mode by monitoring the transitions of deprotonated ions [M-H]^−^. For each analyte, two transitions were monitored: one for quantification and the other for confirmation. All the MS/MS parameters are described in Table 1. Procedural blank samples with ultrapure water in place of urine were collected, extracted, and analyzed by HPLC-MS/MS following the same protocol. In all processed blanks, we did not observe BPA contamination above the method limit of detection (LOD). All solvents and reagents (i.e., acetonitrile, acetone, ammonium acetate, and chloroform) were from VWR International (Radnor, PA, USA). All aqueous solutions were prepared with ultrapure water, Millipore Milli-QTM. Analytical standard compounds were purchased from Sigma-Aldrich (Milan, Italy). The method was based upon previously published methods with slight modifications [23]. To check method performances, we validated our procedure by verifying quality parameters according to Eurachem guidelines [24]. We checked for selectivity, sensitivity, linearity, accuracy, and repeatability. In particular, method sensitivity LOD was 0.0035 ng mL^−1^ for BPA and 0.0030 ng mL^−1^ for BPS.

#### 2.3.6. Creatinine 

Urinary creatinine was determined to normalize the excretion rate of all the aforementioned urinary biomarkers as previously described [25].

### 2.4. Statistical Analysis

Data were expressed as mean ± SD or counts and percentages. As the statistical distribution of the quantitative parameters was found to be non-Gaussian (Kolmogorov–Smirnov test), non-parametric tests were used to assess between-group differences (Mann–Whitney U-test). For qualitative data, groups were compared using a chi-square or Fisher’s exact test, as appropriate. A two-sided *p*-value < 0.05 was considered to indicate statistical significance. To further analyze possible sources of BP exposure, non-parametric correlations (Spearman correlations) were performed between BP levels and three groups of independent variables: (1) maternal exposure: lifestyle habits in the last months of pregnancy (i.e., daily consumption of food and beverages in plastic packaging or daily use of a microwave and dishwasher), smoking habits, anthropometric characteristics (i.e., weight and BMI composition pre-pregnancy and at the end of pregnancy), age, level of education, and nationality; (2) neonatal hospital procedures, including medical check-ups, oral glucose administration in the first three days of life, and drug administration during delivery (because both were administered from a non-BP-free container); and (3) neonatal exposure: pacifier and formula administration in the first three days of life. All analyses were carried out including only those subjects with no missing data (i.e., complete case analysis).

## 3. Results

Table 2 describes the characteristics of the study population (newborns and mothers). For this pilot study, 200 mothers with their babies were enrolled, but only 134 subjects followed all the selection criteria and were eligible. In fact, in addition to the selection criteria, about 33% of enrolled mothers/babies were excluded for three other main reasons:

1. Not-fully completed information (questionnaire and infant data);
2. Insufficient urine samples (mothers: <30 mL/babies: <5 mL);
3. Withdrawal of informed consent.

The newborn sample was homogenous in terms of height, weight, and cranial circumference (Table 2, part A). About 80% of the babies were breastfed, with only 27% of babies (*n* = 36) receiving infant formula as extra integration before being discharged from the hospital. Moreover, about 30% of newborns were administered oral glucose as extra integration and/or pacifiers. Sixteen babies (12%) required respiratory procedures, while 24% of newborns (n = 32) were diagnosed with potentially pathological conditions after general medical check-up. The sample of mothers was homogenous according to age, height, weight, and BMI variables. Among mothers (*n* = 134), 94% were European while 6% were classified as other nationalities (non-European). The education level was low (i.e., primary and middle school) in 14.9% of subjects, while it was medium and high (secondary school and above) in 38% and 47.1% of subjects, respectively. Fifteen mothers (11%) reported being active smokers during pregnancy, whereas twenty-one (15.7%) were passive smokers. 

Table 3 reports the BP concentrations in the study population, split up for newborn and mother groups. On average, newborns had BP levels twice as high as mothers, except for free BP forms (both for free BPA and free BPS). The determination of conjugated BPs was performed by an indirect procedure based on the enzymatic cleavage of the glucuronic acid/sulfuric acid moiety from the phenol group(s) of BPs. A recent report [26] evidenced the possibility of underestimating the real quantity of bound BPs because of different yields of enzymatic reactions together with the chance of the formation of intermediates. In order to evaluate reaction yields, we performed the enzymatic incubation for different times (from 4 to 12 h), confirming that a stable result was reached at 12 h. We also checked for the presence of partially cleaved BP metabolites by high-resolution mass-spectrometry analysis (data not shown), excluding this likelihood.

Because the biological variables had non-Gaussian distributions, non-parametric tests (Mann–Whitney test and Spearman’s rho correlations) were performed. In the mother group, no BP differences were detected in terms of anthropometric characteristics (i.e., weight and BMI composition pre-pregnancy and at the end of pregnancy), age, level of education, and nationality. Besides, the analysis showed no significant correlation between maternal and neonatal BP concentration levels. 

**Figure 1:**
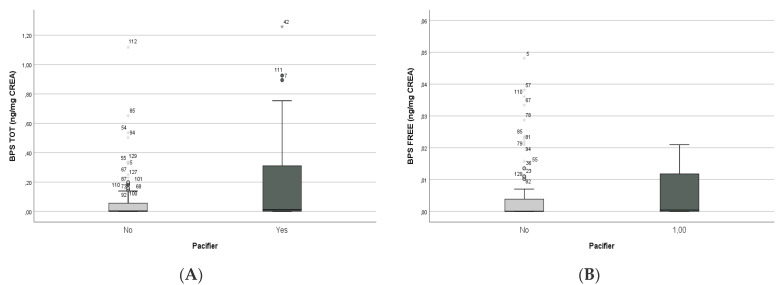
Non-parametric correlations between pacifier use and BPS total (**A**) and free BPS (**B**) concentrations in newborns.

In the newborn group, BP analysis revealed no significant differences (Mann–Whitney U-test) between mothers’ lifestyle habits, such as daily consumption of food and beverages in plastic packaging (BPA total *p* = 0.6; BPS total *p* = 0.2), daily use of a microwave and dishwasher (BPA total *p* = 0.5; BPS total *p* = 0.8), or make-up use (BPA total *p* = 0.7; BPS total *p* = 0.08). In newborns, the most important correlation was found between pacifier use and BPS concentrations, in terms of both total BPS (Mann–Whitney U-test, *p* = 0.004; Spearman’s rho = 0.182, *p* = 0.035—Figure 1A) and free BPS concentrations (Mann–Whitney U-test, *p* = 0.003; Spearman’s rho = 0.190, *p* = 0.03—Figure 1B).

**Figure 2:**
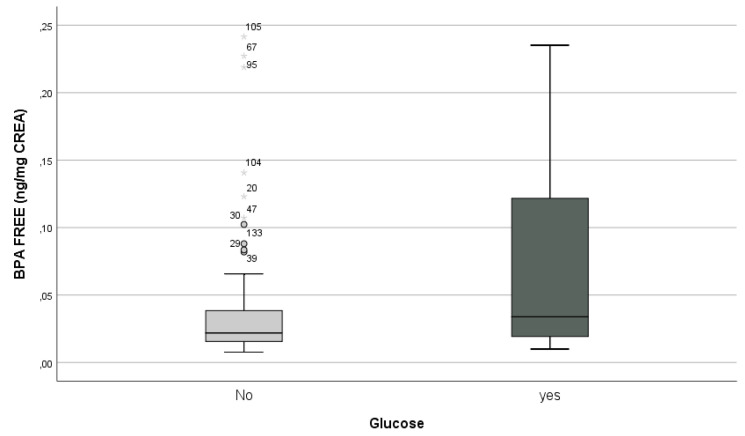
Non-parametric correlations between oral glucose administration and free BPA levels in newborns.

A significant correlation was also found between oral glucose administration and concentration levels of free BPA (Mann–Whitney U-test, *p* = 0.003; Spearman’s rho = 0.182, *p* = 0.035—Figure 2). 

**Figure 3:**
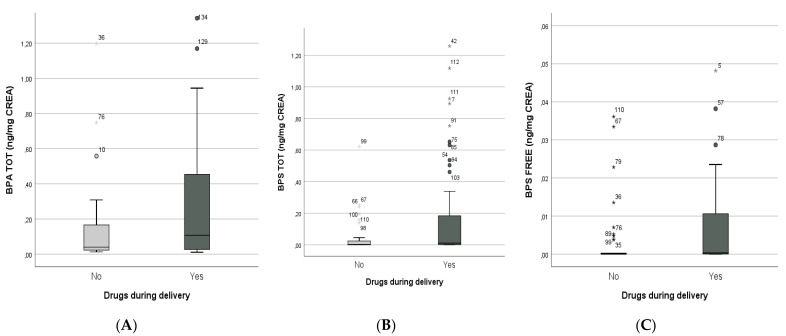
Correlations between drugs administered during delivery and BPA total (**A**), BPS total (**B**), and free BPS (**C**) concentrations in newborns.

Subsequently, positive correlations were found between drugs administered during delivery and total BPA (Mann–Whitney U-test, *p* = 0.05; Spearman’s rho = 0.146, *p* = 0.04—Figure 3A), total BPS (Mann–Whitney U-test, *p* = 0.001; Spearman’s rho = 0.198, *p* = 0.001—Figure 3B), and free BPS (Mann–Whitney U-test, *p* = 0.05; Spearman’s rho = 0.170, *p* = 0.05—Figure 3C) concentrations. 

**Figure 4:**
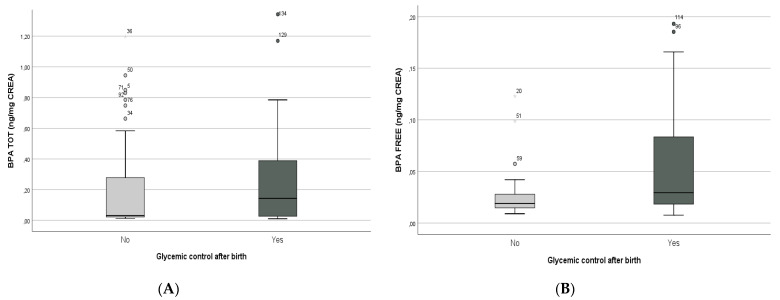
Correlations between glycemic control after birth and BPA total (**A**) and free BPA (**B**) concentrations.

In further analyses, despite the exclusion of pre-term births from the population study, positive correlations were found between BPA total (Mann–Whitney U-test *p* = 0.001; Spearman’s rho = 0.172, *p* = 0.001) and free BPA (Mann–Whitney U-test *p* = 0.001; Spearman’s rho = 0.256, *p* = 0.003) concentrations and glycemic control after birth (Figure 4A,B, respectively).

## 4. Conclusions

The toxic properties of BPs are well-known, and their widespread presence in the environment poses a substantial risk to human health. Thus, assessing BP environmental distribution and the possible presence of these compounds in newborns and their mothers’ biological fluids represents an urgent need to prevent BP-related diseases. BPs are particularly dangerous for children and pregnant women, who are generally highly vulnerable [13]. In fact, because glucuronidation is less efficient in newborns, their internal dose of BPs may be higher and more persistent compared to the general population. As previously cited, the European Food Safety Authority has recently called for a reduction of BPA in food and drink packaging, recommending the replacement of BPA-containing epoxy resins with plastics containing alternative BPs, such as BPS or BPF. For these compounds, no regulation is available yet, and recent data have shown that BPS may also exert genotoxic and estrogenic activities, similar to BPA [19]. All these lines of evidence converge in the reasonable fear that BPS could rapidly become a major health concern. 

In this study, we measured BP concentration levels in a group of newborns and their mothers, and assessed factors contributing to BP risk exposure during prenatal and neonatal life. Based on our findings, we can draw two main conclusions and two strong hypotheses: 

Conclusion 1: Neonatal routine procedures may expose newborns to BPs. The observation that newborns receiving oral glucose after birth displayed high BPA levels, especially in its toxic free form, suggests that oral glucose administration through non-BPA-free containers and syringes should be avoided. Besides, drug administration to the mother via non-BPA-free containers and syringes during delivery is also positively correlated with higher levels of both total BPS and BPA concentrations in newborns. Using syringes to administer dextrose solutions or other parenteral products to newborns is a widespread practice, and these results show the risk associated with such a practice [27]. Thus, our findings advance, once again, the need for extending EU regulations to non-alimentary or medical sources of BP contamination. They also call for further studies on BP exposure in newborns in order to identify which devices and procedures are best suited to minimize BP intake in this extremely vulnerable population. In fact, medical devices are a specific product category in which BP may be present, and preventive actions must be adopted to reduce or even eliminate this exposure source [28].

Conclusion 2: Newborn exposure to BPS can still occur through pacifiers. The observation that the use of pacifiers was associated with higher levels of both total and free BPS indicates the urgent need of in-depth analyses and regulations of BPA alternatives such as BPS. The vast majority of regulations have been directed towards BPA-containing plastics, while they have failed to take into account other plastic products containing alternative BPs. In fact, BPS was regarded as a “safe” alternative to BPA because of its greater stability against high temperatures and resistance to sunlight compared to BPA. No previous epidemiological study has explored the developmental effects of BPS, but in vitro studies have shown that BPS can bind to estrogenic receptors (ERs) and drive estrogen-induced gene transcription [29,30]. Furthermore, studies in zebrafish have shown that BPS exposure can alter the homeostasis of sex steroid hormones and disrupt reproduction or development, miming BPA exposure [31,32]. Current knowledge on the impact of BPS exposure is limited, but, on these bases, further studies are advocated to deepen knowledge of human BPS intake and exposure, focusing more on newborn health effects [33].

**Hypothesis** **1:** *Lifestyle habits of the mother in the last month of pregnancy do not influence BP exposure. We showed that the lifestyle habits of pregnant women in the last month of pregnancy did not cause significant changes in newborns’ BP levels. In fact, no stratification effect was detected due to lifestyle, food habits, education level, or work. This could be explained by analyzing some pregnancy-related factors, such as more attention in the diet habits of pregnant women (no consumption of plastic-free or precooked food) or lifestyle habits.*

**Hypothesis** **2:** *BP levels could exacerbate health conditions at birth and increase the duration of hospitalization. We showed that newborns with higher levels of free BPA/BPS had higher risk of diseases at birth and higher duration of hospitalization. Further investigation is needed to elucidate if specific conditions are related to this association and how they are related. Nevertheless, BPs’ toxic effects may play a role in worsening a newborn’s clinical status and recovery time through hormonal/metabolic impairment, resulting in a longer hospital stay.*

Strengths and limitations of this study: Strengths of our study include high-sensitivity biological analyses and the planning and sampling of the subject immediately after birth (within the first three days of the newborn’s life). One of the biggest limitations is that we planned a cross-sectional study so we are only able to describe the situation. Besides, the study analyzed a small sample, and the information about lifestyle habits is limited to the last month of pregnancy. 

This is the first report of newborn BP contamination due to widely employed non-alimentary products designed for newborn care. Moreover, our study points to a central role of hospitalization procedures and neonatal devices as primary sources of BP exposure. We also show that lifestyle and alimentary habits, especially in the last month of pregnancy, did not influence BP risk exposure. Despite this, in general, harmful behaviors could negatively impact newborn health conditions, and it is necessary to improve preventive strategies to counteract this trend. Finally, we showed the potential pathological effects of BP exposure on a newborn’s clinical status. Further studies are advocated in order to clarify both the impact of other BP forms on humans and the potential sources and consequences of BP exposure during neonatal and childhood life.

## Figures and Tables

**Table 1:** MS/MS parameters for bisphenol A (BPA) and bisphenol S (BPS) analyses.

Analyte	Molecularion (*m/z*)	Fragmention (*m/z*)	Declustering Potential (V)	Entrance Potential (V)	Collision Energy (V)	Collision Cell Exit Potential (V)
**BPA**	227.0	212.0	−79	−9	−23	−14
	227.0	133.0	−79	−9	−30	−14
**BPA-d16**	241.0	223.0	−62	−8	−25	−11
	241.0	142.0	−62	−8	−33	−18
**BPS**	249.0	108.0	−75	−3	−35	−13
	249.0	92.0	−75	−3	−38	−13
**BPS-d8**	257.0	112.0	−120	−7	−34	−14
	257.0	96.0	−120	−7	−35	−14

**Table 2:** Physical characteristics of the newborn sample at birth and BP (i.e., BPA and BPS) concentrations (part A); Characteristics of mother population (part B).

PART A	NEWBORN GROUP (*n* = 134)		
**Sex**	male	84 (62.7%)	
	female	50 (37.3%)	
**Height at birth (cm) (Mean ± SD)**	52.9 ± 1.3		
**Weight at birth (kg) (Mean ± SD)**	3.3 ± 0.4		
**Cranial circumference (cm) (Mean ± SD)**	38.3 ± 1.6		
**Breast-fed N (%)**	111 (82.8%)		
**Infant formula dispensed N (%)**	36 (26.9%)		
**Glucose dispensed N (%)**	38 (28.4%)		
**Pacifier dispensed N (%)**	42 (31.3%)		
**Respiratory procedures N (%)**	16 (11.9%)		
**General medical check-up N (%)**	85 (63.4%)		
**Diseases at birth N (%)**	32 (23.9%)		
**PART B**	**MOTHER GROUP (*n* = 134)**		
**Age (Mean ± SD)**	33.9 ± 4.7		
**Height (cm) (Mean ± SD)**	164.1 ± 7.1		
**Weight (kg) (Mean ± SD)**	Pre-pregnancy	64.4 ± 13.9	
	End of pregnancy	76.8 ± 13.7	
	Δ	12.3 ± 4.8	
**BMI (Mean ± SD)**	Pre-pregnancy	23.9 ± 4.9	
	End of pregnancy	28.5 ± 4.8	
**Nationality N (%)**	European	126 (94%)	
	Others	8 (6%)	
**Education level N (%)**	Low level	20 (14.9%)	
	Medium level	51 (38%)	
	High level	63 (47.1%)	
**Occupation N (%)**	Yes	114 (85.1%)	
	No	6 (4.5%)	
	Others	14 (10.4%)	
**Living place N (%)**	Rural	40 (29.9%)	
	Suburban	51 (38.1%)	
	Urban	43 (32.1%)	
**Smoking habits N (%)**	No	98 (73.1%)	
	Passive (Average exposure time: 3 h)	21 (15.7%)	
	Yes	15 (11.2%)	
		<10 cig/die	8 (53.3%)
		>10 cig/die	7 (46.7%)
**Delivery N (%)**	Vaginal	81 (60.4%)	
	Caesarian	46 (34.3%)	
	Vacuum	7 (5.2%)	

**Table 3:** BP levels (BPA and BPS) in the newborn and mother groups.

BP Levels (ng/mL) (Mean ± SD; C.I. Range)	Newborn	Mother	BP Levels (ng/mg _CREA_) (Mean ± SD; I.C. Range)	Newborn	Mother
**Total BPA**	0.13 ± 0.3 [0.02/0.74]	0.15 ± 0.23 [<LOD/0.62]	Total BPA	0.48 ± 1.13 [0.02/2.5]	0.24 ± 0.43 [<LOD/0.9]
**Conjugated BPA**	0.11 ± 0.3 [0.01/0.6]	0.13 ± 0.2 [<LOD/0.53]	Conjugated BPA	0.41 ± 1.05 [>0.004/2.3]	0.2 ± 0.35 [<LOD/0.75]
**Free BPA**	0.01 ± 0.37 [<LOD/0.62]	0.02 ± 0.06 [<LOD/0.13]	Free BPA	0.07 ± 0.09 [<0.01/0.25]	0.03 ± 0.16 [<LOD/0.1]
**Total BPS**	0.09 ± 0.2 [<LOQ/0.6]	0.01 ± 0.03 [<LOQ/0.06]	Total BPS	0.2 ± 0.53 [<LOD/0.1]	0.04 ± 0.15 [<LOD/0.1]
**Conjugated BPS**	0.08 ± 0.17 [<LOD/0.5]	0.005 ± 0.04 [<LOD/0.05]	Conjugated BPS	0.15 ± 0.5 [<LOD/0.9]	0.02 ± 0.14 [<LOD/0.04]
**Free BPS**	0.01 ± 0.04 [<LOD/0.05]	0.004 ± 0.002 [<LOD/0.02]	Free BPS	0.02 ± 0.07 [<LOD/0.01]	0.02 ± 0.06 [<LOD/0.03]
**Creatinine (CREA) (mg/L)**	MOTHER	0.66 ± 0.5 [0.1/1.9]			
	NEWBORN	0.8 ± 0.4 [0.1/1.5]			

## Data Availability

The data presented in this study are available on request from the corresponding author. The data are not publicly available due to the participant’s privacy protection.
